# Chemical Composition and Antibacterial Activity of the Essential Oils of *Callistemon citrinus* and *Callistemon viminalis* from South Africa

**DOI:** 10.3390/molecules14061990

**Published:** 2009-06-02

**Authors:** Opeoluwa O. Oyedeji, Oladipupo. A. Lawal, Francis. O. Shode, Adebola. O. Oyedeji

**Affiliations:** 1 School of Chemistry, University of KwaZulu-Natal, Westville Campus, South Africa; 2 Department of Chemistry, University of Zululand, KwaDlangezwa 3886, South Africa

**Keywords:** *Callistemon citrinus*, *Callistemon viminalis*, Myrtaceae, essential oil composition, antibacterial activity

## Abstract

The chemical composition and the antibacterial activity of the essential oils obtained by hydrodistillation from the leaves of *Callistemon citrinus* and *Callistemon viminalis* were analyzed by GC and GC/MS. Twenty-four and twelve components were identified for *C. citrinus* and *C. viminalis*, representing 92.0% and 98.3% of the total oils. The major components of *C. citrinus and C. viminalis* were 1,8-cineole (61.2% and 83.2%) and α-pinene (13.4% and 6.4%), respectively. The *in vitro* antibacterial activity of the essential oils was studied against 12 bacteria strains using disc diffusion and broth microdilution methods. The oils exhibited strong zone of inhibitions against some bacteria such as *S. faecalis* (20.3-24.0 mm), both strains of *S. aureus* (23.0-26.3 mm), *B. cereus* (17.3-19.0 mm) and *S. macrcesens* (11.3-23.7 mm) when compared to standard antibiotics gentamycin and tetracycline used as controls. Expect for *P. aeruginosa* and *S. macrcescens*, the MIC values of both essential oils ranged from 0.31-2.50 mg/mL.

## 1. Introduction

The genus *Callistemon* R. Br. (commonly known as bottlebrush) belongs to the family Myrtaceae and comprises over 30 species. They are woody aromatic trees or shrubs (ca. 0.5 m to 7 m tall) widely distributed in the wet tropics, notably Australia, South America and tropical Asia, but are now spread all over the world. *Callistemon* species have attractive narrow foliage and white papery bark. The leaves of *Callistemon* species are lanceolate (ca. 3-6 mm wide and 40-70 mm long) in arrangement and very aromatic. The flowers are borne in spikes of about 40-150 mm long with prominent red stamens. Petals are greenish or pale coloured, tiny, inconspicuous and in some cases deciduous [1,2]. 

*Callistemon* species are used for forestry, essential oil production, farm tree/windbreak plantings, degraded-land reclamation and ornamental horticulture, among other applications [1]. In China callistemon species, especially *C. viminalis*, are used in Traditional Chinese Medicine pills for treating hemorrhoids [3]. *Callistemon* are also used as weed control [4] and as bioindicators for environmental management [5]. Previous phytochemical investigations of members of this genus resulted in the identification of *C*-methyl flavonoids, triterpenoids and phloroglucinol derivatives [6,7,8,9,10]. Furthermore, piceatannol and scirpusin B isolated from the stem bark of *C. rigidus* from Japan, showed inhibitory effects on mouse α-amylase activity [11]. In addition, antimicrobial, anti-staphylococcal, antithrombin, repellent and nematicidal activities as well as larvicidal and pupicidal values have been reported for the genus [12,13,14,15,16,17]. 

In the flora of South Africa, *Callistemon* species are grown as garden, street trees or ornamental plants due to their decorative flowers. *C. citrinus* (Curtis) Skeels (syn: *Metrosideros citrina* Curtis; commonly known as crimson or lemon bottlebrush) is a handy medium shrub to large tree (ca. 5-7 m tall). *C. citrinus* is the most widely cultivated member of the genus *Callistemon*. The bright red flower spikes of *C. citrinus* are very rich in nectar and attract many birds [1,6].

*C. viminalis* (Sol. ex Gaertner) G. Don ex Loudon (commonly known as weeping bottlebrush) is a small tree or shrub with pendulous foliage, although some forms are more pendulous than others. It reaches a height of about 4 m in its natural habitat, but is usually smaller in cultivation, particularly in temperate areas [1,6]. 

Chemical studies of the essential oils of *C. citrinus* and *C. viminalis* from Australia, Egypt, India, Pakistan and Reunion Island have been previously reported [18,19,20,21,22]. 1,8-Cineole (47.9-82.0%) was the predominant constituent of the oils. Other significant components included α-pinene, β-pinene, myrcene, limonene, linalool and menthyl acetate. In addition, a few reports on the biological activities of *C. citrinus* and *C. viminalis* essential oils reveal anthelmintic and anti-quorum sensing activities [23,24,25]. To the best of our knowledge, there are no reports on the chemical composition of *Callistemon* species growing in South Africa, so the present paper reports for the first time the volatile constituents and antibacterial activity of the essential oils of *C. citrinus* and *C. viminalis* from South Africa.

## 2. Results and Discussion

The yields of the oils obtained from the hydrodistillation of the leaves of *C. citrinus* and *C. viminalis* were 1.2% and 0.9% (w/w), respectively. Table 1 lists the components identified in the essential oils with their percentage composition and relative retention indices. Twenty-four constituents were identified and quantified in the oil of *C. citrinus,* representing 92.0% of the total oil. The major components were 1,8-cineole (61.2%), α-pinene (13.4%) and β-pinene (4.7%). In the oil of *C. viminalis*, twelve constituents (98.3%) were identified and quantified. The major compounds were 1,8-cineole (83.2%), α-pinene (6.4%) and α-terpineol (4.9%). In both cases, the most abundant constituents were the oxygenated monoterpenes (70.3 -90.1%) with 1,8-cineole (61.2 - 83.2%) constituting the bulk of the oils, when compared to monoterpene hydrocarbons (7.7 - 21.6%). α−Pinene (13.4 - 6.4%), α-terpineol (4.2 - 4.9%) and β-pinene (0.9 - 4.7%) were other prominent compounds identified in the oil. Furthermore, components like α-terpinene, linalool, trans-pinocarveol, terpine-4-ol and geraniol were identified in trace amounts in both *C. citrinus* and *C. viminalis* essential oils in this study. 

**Table 1:** Chemical Constituents of essential oil of *C. citrinus* and *C. viminalis* leaves.

Compound	RI^a^		Percentage composition		
			*C. citrinus*		*C. viminalis*
*α*-thujene	933		t		-
*α*-pinene	937		13.4		6.4
camphene	948		t		-
*β*-pinene	976		4.7		0.9
myrcene	989		-		t
*α*-phellandrene	1004		2		-
*α*-terpinene	1016		0.9		0.4
1,8-cineole	1027		61.2		83.2
*Z*-(*β*)-ocimene	1036		t		-
*α*-terpinolene	1054		0.6		-
linalool	1096		0.8		0.5
fenchol	1109		0.1		-
*trans*-pinocarveol	1138		0.3		0.9
pinocarvone	1154		-		t
terpinen-4-ol	1168		2		0.6
crypton e	1179		t		-
*α*-terpineol	1187		4.2		4.9
*trans*-carveol	1220		0.1		t
citronellol	1228		0.2		-
carvone	1247		t		-
geraniol	1244		0.9		0.5
eugenol	1361		0.2		-
geranyl acetate	1381		0.3		-
spathulenol	1570		t		-
caryophyllene oxide	1583		t		-
ledol	1606		0.1		-
Monoterpene hydrocarbons			21.6		7.7
Oxygenated monoterpenes			70.3		90.1
Sesquiterpene hydrocarbons			-		-
Oxygenated sesquiterpenes			0.1		-
Total identified			92		98.3

RI^a^ = Kovat index relative to C_9_-C_24_ *n*-alkanes on HP-5 column; t = trace (< 0.05%)

Although, the essential oil compositions of *C. citrinus* and *C. viminalis* from different countries have been studied [18,19,20,21,22], there are differences in the yield and constituents of the oils, which could be attributed to difference in generic and geographical/environmental conditions. The abundance of 1,8-cineole in the essential oils of *C. citrinus* and *C. viminalis* makes them similar to those obtained in all the previous studies from India, Australia, Egypt and Reunion Island samples, but a key difference in the oils lies in the relative quantities of α-pinene, β-pinene limonene, linalool and α-terpineol. In contrast to the report concerning the analysis of the essential oil of *C. viminalis* growing in Egypt [21], our study on South African species showed quantitative differences. In the oil of *C. viminalis* from Egypt, 1,8-cineole represented 47.9%% of the total oil, while in the South African species, it was 83.2%. In addition, linalool (13.0%) and limonene (10.9%), which appeared as major constituents in the Egyptian species were present in low concentration (linalool 0.5%) or were absent (limonene) in the South Africa species.

The inhibition zone of the disc diameters (IZ) and minimum inhibitory concentrations (MICs) of the essential oils of *C. citrinus* and *C. viminalis* against the microorganisms tested are shown in Table 2. The results obtained from the agar disc diffusion method for the essential oils revealed *S. aureus* (ATCC 3983) to be the most sensitive microorganism with the largest inhibition zones (26.3 and 25.0 mm for *C. citrinus* and *C. viminalis*, respectively), while the smallest inhibition zones were exhibited by *E. coli* and *K. pneumoniae* (13.3 mm) for *C. citrinus* and *S. marcescena* and *P. aeruginosa* (11.3 and 10.3) mm for *C. viminalis* respectively. 

**Table 2:** Antibacterial activity of the essential oils of *C. citrinus* and *C. Viminalis.*

Micro organisms	*C. citrinus*		*C. viminalis*		Gentamycin		Tetracycline	
	IZ	MIC	IZ	MIC	IZ	MIC	IZ	MIC
*B. cereus* (ATCC 10702)	17.3 ± 1.5	1.25	19.0 ± 1.7	0.63	14.0 ± 2.0	0.63	13.3 ± 2.0	1.25
*B. pumilus (*ATCC 14884)	13.7 ± 1.5	1.25	15.3 ± 1.2	1.25	13.3 ± 2.1	1.25	14.0 ± 1.5	1.25
*S. aureus* (ATCC 3983)	26.3 ± 2.0	0.31	24.7 ± 1.2	0.08	17.3 ± 1.2	0.31	18.7 ± 2.6	0.31
*S. aureus* (ATCC 6538)	25.0 ± 1.5	0.63	23.0 ± 1.7	0.63	14.4 ± 1.5	0.63	ND	0.31
*S. faecalis* (ATCC 29212)	24.0 ± 1.0	0.63	20.3 ± 2.	0.63	16.0 ± 2.0	1.25	ND	ND
*E. cloacae* (ATCC 13047)	18.3 ± 1.5	1.25	17.7 ± 2.5	0.63	12.6 ± 0.6	2.5	13.0 ± 0.6	2.5
*E. coli* (ATCC 4983)	13.3 ± 1.2	1.25	14.3 ± 1.5	2.5	21.3 ± 1.5	0.16	23.0 ± 1.7	0.31
*K. pneumoniae* ATCC 2982)	13.3 ± 1.7	2.5	14.3 ± 0.6	2.5	23.7 ± 1.5	0.08	17.6 ± 1.5	0.63
*P. vulgaris* (ATCC 6830)	17.0 ± 1.7	2.5	16.0 ± 0.0	2.5	21.3 ± 1.2	0.31	6.0 ± 0.0	ND
*P. vulgaris* (CSIR 0030)	18.3 ± 1.7	2.5	18.3 ± 1.5	1.25	6.0 ± 0.0	5	6.0 ± 0.0	5
*P. aeruginosa* (ATCC 7700)	15.3 ± 2.1	2.5	10.3 ± 0.6	5	20.7 ± 1.2	0.63	14.7 ± 0.6	0.63
*S. marcescens* (ATCC 9986)	23.7 ± 0.6	0.63	11.3 ± 1.2	5	7.3 ± 0.0	2.5	15.7 ± 1.5	0.63

IZ = Zone of inhibition; MIC = minimum inhibitory concentrations; Dose: 5 mg/mL; Disc diameter; 6 mm. Values are the mean ± S.D of the mean; ATCC = American Type Culture Collection; ND = Not Determined

Furthermore, the MIC values showed *S. aureus* (ATCC 3983) having the lowest MIC value (0.08 mg/mL) for both oils and the highest MIC was 5.00 mg/mL for the oil of *C. viminalis* for *P. aeruginosa* and *S. marcescena*, respectively. When compared with standard antibiotics (gentamycin and tetracycline), the essential oils showed a weak to moderate range inhibition zones (10.3 ± 0.2 to 26.3 ± 2.0 mm) vs. the standard antibiotics (6.0 ± 0.0 to 23.7 ± 1.5 mm).

Generally, the essential oils can be said to be of medicinal potential because of the high concentration of 1,8-cineole which is a marker for medicinal essential oil classification. The bacteria used are known to be mostly human pathogens whose effects are noticeable in skin, intestinal and respiratory infections. The antibacterial tests also reveals both oils have a broad spectrum antimicrobial activity against all the tested organisms. This observation is particularly noteworthy because plant extracts are known to be more active against Gram +ve than Gram -ve bacteria and these extracts exhibited notable antibacterial activity against all the bacteria species tested [28,29,30,31]. However, Gram-positive bacteria were more susceptible than Gram-negative bacteria. The antibacterial activity showed by the essential oils of *C. citrinus* and *C. viminalis* could be attributed to the presence of some major components such as 1.8-cineole, α-pinene and α-terpineol, along with other components in lower amount such as, β-pinene and linalool, which were already known to exhibit antimicrobial and bacteriostatic activities [31,32,33,34] nevertheless, the presence of minor component could also play a role in the biological activity. 

## 3. Materials and Methods

### 3.1. Plant materials

The *Callistemon viminalis* and *Callistemon citrinus* plant materials were collected from Durban and Johannesburg, both in the Province of KwaZulu-Natal, South Africa. The taxonomic identification of the plant materials was confirmed by a senior plant taxonomist, Dr S.J. Siebert of the Department of Botany, University of Zululand, KwaDlangezwa. Voucher specimens [OOO 13 & 14 (ZULU)] were deposited at the University of Zululand, Herbarium.

### 3.2. Extraction of essential oil

Fresh matured leaves (300 g each) of *Callistemon viminalis* and *Callistemon citrinus* samples were hydrodistilled for 3 h in a Clevenger-type apparatus [35,36,37]. The resulting oils were collected, preserved in a sealed amber glass sample tube and stored at 4^o^C under refrigeration until analysis. 

### 3.3. GC analysis

The GC analyses of the volatile oils were carried out on a Hewlett Packard HP 6820 Gas Chromatograph equipped with FID detector and HP-5 column (60 m x 0.25 mm id, 0.25 µm film thickness) with a 1:25 split ratio. The oven temperature was programmed from 50 ^o^C (after 2 min) to 240 ^o^C at 5 ^o^C/min and the final temperature was held for 10 min. Injection and detector temperatures were 200^o^C and 240 ^o^C respectively. Hydrogen was the carrier gas. Diluted oil (0.5 µL) was injected into the GC. Peaks were measured by electronic integration. *n*-Alkanes were run under the same condition for Kováts indices determination.

### 3.4. GC/MS analysis

GC-MS analyses of the oils were performed on a Hewlett Packard HP 6890 Gas Chromatograph interfaced with a Hewlett Packard 5973 mass spectrometer system equipped with a HP 5-MS capillary column (30 m x 0.25 mm id, film thickness 0.25 µm). The oven temperature was programmed from 70- 240 ^o^C at the rate of 5^o^C/min. The ion source was set at 240 ^o^C and electron ionization at 70Ev. Helium was used as the carrier gas (1mi/min). The split ratio was 1:25 with the scan range of 35 to 425 amu. 1.0 µL of diluted oil in hexane was manually injected into the GC/MS.

### 3.5. Identification of compounds

The components of the oils were identified base on the comparison of their retention indices and mass spectra with those standards, the Wiley Library of Mass Spectra database of the GC/MS system and published data [38,39,40].

### 3.6. Antibacterial assay

The essential oils were tested against 12 reference bacterial strains obtained from the Department of Biochemistry & Microbiology, University of Fort Hare, Alice. Gram-positive bacteria: *Bacillus cereus* (ATCC 10702), *Bacillus pumilus* (ATCC 14884), *Staphylococcus aureus* (ATCC 3983), *Staphylococcus aureus* (ATCC 6538) and *Streptococcus faecalis* (ATCC 29212). Gram-negative strains: *Enterobacter cloacae* (ATCC 13047), *Escherichia coli* (ATCC 4983), *Kiebsiella pneumoniae* (ATCC 2983), *Proteus vulgaris* (ATCC 6830), *Proteus vulgaris* (CSIR 0030), *Pseudomonas aeruginosa* (ATCC19582) and *Serratia marcescens* (ATCC 9986). The stock cultures were maintained at 4 ^o^C in Mueller-Hinton agar (MHA) (Oxoid). 

#### 3.6.1. Agar disk diffusion

The essential oils were tested for antibacterial activity by the agar disc diffusion method according to Kiehlbauch *et al*. [35]. The microorganisms were grown overnight at 37 ^o^C in 20 mL of Mueller-Hinton broth (Oxoid). The cultures were adjusted with sterile saline solution to obtain turbidity comparable to that of McFarland no. 5 standard (1.0 x 10^8^) CFU/mL. 90 mm Petri dishes (Merck, South Africa) containing 12 mL of sterilized Mueller-Hinton agar (Oxoid) were inoculated with these microbial suspensions. Sterile Whatman No.1 (6 mm) discs papers were individually placed on the surface of the seeded agar plates and 10 µL of essential oil in dimethylsulfoxide (DMSO) was applied to the filter paper disk. The plates were incubated at 37 ^o^C for 24 h and the diameter of the resulting zones of inhibition (mm) of growth was measured. All tests were performed in triplicates. Gentamycin and tetracycline were used as positive controls, while hexane and DMSO served as negative controls.

#### 3.6.2. Minimum inhibitory concentration

The minimum inhibitory concentration (MIC) of the essential oils was determined using 96-well microtitre dilution method as described by Oyedeji and Afolayan, and Eloff [42,43]. Bacterial cultures were incubated in Müller-Hinton broth overnight at 37 ^o^C and a 1:1 dilution of each culture in fresh MH broth was prepared prior to use in the micro dilution assay. Sterile water (100 μL) was pipetted into all wells of the microtitre plate, before transferring 100 μL of essential oil in DMSO. Serial dilutions were made obtain concentrations ranging from 10 mg/mL to 0.078 mg/mL. One hundred μL of bacterial culture of an approximate inoculum size of 1.0 x 10^8^ CFU/mL was added to all well and incubated at 37 ^0^C for 24h. After incubation, 40 μL of 0.2 mg/mL *p*-iodonitotetrazolium violet (INT) solution was added to each well and incubated at 37 ^o^C. Plates were examined after about 30-60 min. of incubation. Microbial growth is indicated by the presence of a reddish colour which is produced when INT, a dehydrogenase activity detecting reagent, is reduced by metabolically active micro-organisms to the corresponding intensely coloured formazan. MIC is defined as the lowest concentration that produces an almost complete inhibition of visible micro-organism growth in liquid medium. Solvent controls (DMSO and hexane) and the standard antibiotics gentamycin and tetracycline were included in the assay.
